# Sliding scale HCG trigger yields equivalent pregnancy outcomes and reduces ovarian hyperstimulation syndrome: Analysis of 10,427 IVF-ICSI cycles

**DOI:** 10.1371/journal.pone.0176019

**Published:** 2017-04-25

**Authors:** Vinay Gunnala, Alexis Melnick, Mohamad Irani, David Reichman, Glenn Schattman, Owen Davis, Zev Rosenwaks

**Affiliations:** The Ronald O. Perelman and Claudia Cohen Center for Reproductive Medicine, Weill Cornell Medicine, New York, New York, United States of America; Otto von Guericke Universitat Magdeburg, GERMANY

## Abstract

**Objective:**

To evaluate pregnancy outcomes and the incidence of ovarian hyperstimulation syndrome (OHSS) using a sliding scale hCG protocol to trigger oocyte maturity and establish a threshold level of serum b-hCG associated with optimal oocyte maturity.

**Design:**

Retrospective cohort.

**Setting:**

Academic medical center.

**Patients:**

Fresh IVF cycles from 9/2004–12/2011.

**Intervention:**

10,427 fresh IVF-ICSI cycles met inclusion criteria. hCG was administered according to E2 level at trigger: 10,000IU vs. 5,000IU vs. 4,000IU vs. 3,300IU vs. dual trigger (2mg leuprolide acetate + 1,500IU hCG). Serum absorption of hCG was assessed according to dose and BMI.

**Main outcome measures:**

Oocyte maturity was analyzed according to post-trigger serum b-hCG. Fertilization, clinical pregnancy, live birth and OHSS rates were examined by hCG trigger dose.

**Results:**

Post-trigger serum b-hCG 20–30, 30–40, and 40–50 mIU/mL was associated with reduced oocyte maturity as compared b-hCG >50 (67.8% vs. 71.4% vs. 73.3% vs. 78.9%, respectively, *P*<0.05). b-hCG 20–50 mIU/mL was associated with a 40.1% reduction in live birth (OR 0.59, 95% CI 0.41–0.87). No differences in IVF outcomes per retrieval were seen for varying doses of hCG or dual trigger when controlling for patient age. The incidence of moderate to severe OHSS was 0.13% (n = 14) and severe OHSS was 0.03% (n = 4) of cycles.

**Conclusions:**

Moderate stimulation with sliding scale hCG at trigger and fresh transfer is associated with low rates of OHSS and favorable pregnancy rates. Doses as low as 3,300IU alone or dual trigger with 1,500IU are sufficient to facilitate oocyte maturity.

## Introduction

Major advances in the field of assisted reproductive technology (ART) have occurred over the past two decades. Ovarian hyperstimulation syndrome (OHSS), however, remains one of the most dangerous iatrogenic morbidities associated with ovarian stimulation (OS). The clinical phenomenon of OHSS is primarily due to a supra-physiologic release of VEGF in the context of excessive ovarian response to gonadotropins. While minimizing the risk of OHSS remains a perennial challenge of ART even in the era of the GnRH agonist trigger, modifiable risk factors associated with the development of OHSS include the rate of estradiol (E2) rise, the maximal E2 level reached, and number of small and intermediate size follicles on the day of trigger. [1]. While attentiveness to these risk factors will not eliminate OHSS altogether, they may minimize its incidence.

OHSS can carry significant morbidity, particularly its moderate and severe forms. The actual incidence varies based on published studies, but moderate OHSS has been cited to be as high as 3–10% of all ART cycle, and as high as 20% in high-risk populations [2,3]. The incidence of severe OHSS is estimated to be 0.5–2% of all ART cycles, with a mortality rate of 1 in 400,000 [4]. Severe OHSS may also carry perinatal morbidity with studies suggesting an increased risk of preterm delivery [5,6]. Prevention strategies remain the most prudent approach to reducing or eliminating OHSS. A goal of all ART centers should be to identify patients at risk prior to or during stimulation and adjust their management accordingly. Several measures can be adopted to limit the occurrence of clinically relevant OHSS, including but not limited to lower the starting dose of gonadotropins, step down protocols, antagonist based stimulation protocols, coasting, GnRH agonist (GnRHa) triggers, and cryopreservation of embryos with subsequent freeze-thaw transfer [2,3,7–10].

Another strategy to prevent OHSS is to reduce the dose of hCG trigger [11] [12]. Despite the paradigm shift to use antagonist-based protocols with subsequent GnRHa trigger some literature suggests reduced clinical pregnancy, delivery rates and increased miscarriage rates. In addition, patients with significant hypothalamic suppression, either secondary to prolonged oral contraceptive use or due to endogenous low serum LH levels, may have a sub optimal response to GnRH agonist trigger and therefore data regarding hCG trigger alone remains pertinent [13] [14] [15]. Limited data exists, however, on the lowest threshold dose of hCG required to bring about oocyte maturity with high reproducibility, or whether pregnancy rates are affected by lower serum absorption of hCG. To that end, we queried our clinical database to evaluate whether lowering the dose of hCG at trigger for high responders is an effective practice to induce oocyte maturity while decreasing the risk of OHSS and maintaining high pregnancy rates.

## Materials and methods

### Cycle inclusion/exclusion criteria

The study was approved by the Weill Cornell Medical College Institutional Review Board and the need for patient consent was waived. All IVF cycles performed at the Ronald O. Perelman and Claudia Cohen Center for Reproductive Medicine from September 2004 through December 2011 were reviewed. A total of 21,983 fresh IVF cycles occurred at our center during the study period and were screened for inclusion. Inclusion criteria were defined as any fresh IVF intracytoplasmic sperm injection (ICSI) cycle, which utilized the hCG sliding scale protocol. Inclusion was limited to ICSI cycles so as to allow for absolute assessment of oocyte nuclear maturity. ICSI indications included male factor infertility, frozen/donor sperm, poor semen sample on the day of retrieval (characterized at the discretion of the embryologist), and physician/patient preference. For over two decades we have employed the sliding scale hCG protocol with dose adjustment according E2 levels at trigger: 10,000 IU of hCG for E2 < 1500 pg/mL, 5000 IU for E2 1501–2500 pg/mL, 4,000 IU for E2 2501–3,000 pg/mL, and hCG 3300 IU for E2 > 3,000 pg/mL. A sub-group analysis specific to high responders (E2 > 3,000 pg/mL) compared cycles triggered by low dose hCG (3,300 IU) vs. dual trigger (2mg leuprolide acetate + 1,500 IU hCG) from January 2007 to October 2013. Donor egg recipients and patients who underwent prenatal genetic screening (PGS) or diagnosis (PGD) prior to embryo transfer were excluded from analysis.

### Clinical / Laboratory protocol

Protocols for controlled ovarian stimulation (COS), oocyte retrieval, IVF, and embryo transfer procedures have been described previously [16] [17]. In brief, patients were either down regulated with a GnRH agonist (Luprolide acetate: Abbott Pharmaceuticals North Chicago, IL) followed by gonadotropin stimulation using a combination of FSH and HMG (Follistim: Merck, Whitehouse Station, NJ; Gonal-F: EMD-Serono, Rockland, MA; Menopur: Ferring, Parsippany, NJ) once pituitary suppression was confirmed or were stimulated with gonadotropins until criteria was met for suppression with a GnRH antagonist (Ganirelix Acetate: 0.25mg Organon, Roseland, NJ; Cetrotide 0.25mg: EMD-Serono, Rockland, MA) i.e. either the lead follicle attained 13mm mean diameter or estrogen exceeded 300 pg/mL. Luteal suppression for antagonist-based protocols, when utilized, was achieved with estradiol patches starting 8 to 10 days post-LH surge or by using oral contraceptive pills (OCPs). All protocols were individualized based on patients’ age, weight, results of ovarian reserve testing, and prior response to COS. In general poor responders started with a maximum gonadotropin dose of 450–600 IU while patients at risk for a higher response would administer lower gonadotropin doses at the start of stimulation. All patients were monitored with blood tests and ultrasound frequently during stimulation and gonadotropin doses would be decreased in a step-down fashion if there were signs of hyper-response.

Human chorionic gonadotropin (Profasi: EMD-Serono, Rockland, MA; Novarel: Ferring Pharmaceuticals, Parsippany, NJ; or Pregnyl: Schering-Plough, Kenilworth, NJ) was generally administered when at least 2 follicles reached 17 mm. No patients included in the study received recombinant derived hCG for trigger. On the morning after hCG administration, patients’ blood was drawn and serum analyzed for hCG concentration to ensure adequate absorption (mean time 8–10 hours). All hormone measurements were performed on site at the Center for Reproductive Medicine’s Reproductive Endocrinology Laboratory using a Siemens Immulite 2000 immunoassay system. Oocyte retrieval was performed 35 to 37 hours after hCG trigger. One day following oocyte retrieval, luteal progesterone supplementation was initiated using 50 mg of intramuscular (IM) progesterone. In patients who received a dual trigger, estrogen patch 0.1mg, applied every other day was started with progesterone for luteal support. All oocytes were denuded of surrounding cumulus cells to assess nuclear maturity, with mature (MII) oocytes undergoing ICSI. Embryo transfer was performed on day 3 or day 5 with a Wallace catheter (Marlow/Cooper Surgical, Shelton, CT). The day of embryo transfer (3 vs. 5) and the number of embryos to transfer was decided upon after consultation with the patient and were based on the patient’s clinical history and morphologic markers of embryo development.

### Outcome variables assessed

Demographic characteristics of patients meeting inclusion criteria were collected. These included age, body mass index (BMI), number of embryos transferred and number of prior IVF attempts. Based on prior published literature and our own anecdotal clinical experience of reduced oocyte maturity at post-trigger b-hCG levels < 50 mIU/mL, we analyzed cycles to determine a serum threshold of hCG associated with maximum oocyte maturity [18]. After assessing serum absorption of IM hCG according to dose and BMI we determined which patients were at risk for serum levels below this threshold. IVF cycle characteristics including mature oocyte yield, fertilization rate, number of embryos transferred and pregnancy outcomes were analyzed according to hCG trigger dose. Clinical pregnancy rate was defined as the number of cycles with at least one viable fetus (as evidenced by fetal cardiac activity by ultrasound at seven weeks) per retrieval. Live birth rate was defined as the number of cycles resulting in at least one live born child delivered at ≥ 24 weeks gestation out of all transfers performed. Patients were stratified into SART age categories for analysis. A sub group analysis comparing outcomes between low dose hCG (3,300 IU) vs. dual trigger with GnRH agonist and low dose hCG (2mg leuprolide acetate + 1500 IU hCG) was also performed.

Finally, we evaluated the overall incidence of moderate to severe OHSS with the sliding scale hCG protocol. OHSS was defined clinically by the patient’s provider according to the three tier classification system [7]. In brief, moderate OHSS, which included symptoms of nausea, vomiting, diarrhea and abdominal distention was distinguished from mild OHSS by the presence of ascites following ovarian stimulation. Severe OHSS was characterized by the presence of free fluid in other body cavities (pleural and pericardial), laboratory abnormalities, and/or one of the major complications of OHSS (ARDS, renal failure, thromboembolic phenomena, disseminated intravascular coagulopathy). Patients that were hospitalized due to OHSS or received a drainage procedure (i.e. paractentsis, thoracentesis, culdocentesis) were also defined as having moderate to severe OHSS.

### Statistical analysis

Data analysis was performed with STATA Statistical Software Version 11 (StataCorp LP; College Station, TX). Absorption of hCG according to trigger dose and BMI was analyzed using Spearman-rank correlation coefficients. Multiple comparison test and multivariable logistic regression were used to evaluate oocyte maturity with respect to b-hCG. Differences in demographic characteristics and IVF outcomes according to age and hCG dose administered were calculated with t-test and Chi-square, respectively. In all cases, *P* < 0.05 was considered to be statistically significant.

## Results

Of the 21,983 fresh IVF cycles performed during the study period, 47.4% (10,427 cycles) met inclusion criteria. 5,927 (57%) cycles received an hCG trigger dose of 10,000 IU while 2,485 (24%), 1,354 (13%), and 661 (6%) of the cycles received triggers of 5,000 IU, 4,000 IU, and 3,300 IU respectively. 5,637 (54%) patients had a normal BMI (20–24.9 kg/m^2^) while 20% and 15% of the patients comprised the overweight (BMI 25–29.9 kg/m^2^) and underweight (BMI <20 kg/m^2^) groups, respectively. 10% of the study population was obese with a BMI above 30 kg/m^2^. Of the 1,223 IVF cycles that met inclusion for the sub group analyses of high responders (E2 > 3,000 pg/mL), 883 cycles were triggered with low dose hCG (3,300 IU) and 340 cycles received a dual trigger.

“Table 1” compares oocyte maturity with respect to b-hCG measured the day after hCG trigger. Based on multiple comparison test, the serum b-hCG group > 50 (mIU/mL) was associated with significantly higher oocyte maturity rate when compared to other groups. Because none of the other serum categories (b-hCG 20–30, 30–40, 40–50 mIU/mL) were statistically different from one another, b-hCG > 50 mIU/ml was established as a threshold value for optimal oocyte maturity. After adjusting for age, BMI, number of embryos transferred, and number of prior IVF cycles, serum bHCG on the morning of trigger was found to be an independent predictor of clinical pregnancy rate (adjusted OR 1.61 (95% CI 1.13–2.27), p = 0.008). The 219 cycles in our study population with sub-threshold b-hCG (20–50 mIU/mL) were associated with a 40.1% reduction in live birth (AOR 0.59, 95% CI 0.41–0.87).

**Table 1:** Oocyte maturity with respect to serum b-hCG day after trigger.

	Serum bhCG (mIU/mL)				p value
	20–30 (n = 21)	30–40 (n = 75)	40–50 (n = 123)	>50 (n = 10,003)	
% oocyte maturity	67.8 ± 23.2	71.4 ± 23.6	73.3 ± 18.7	78.9 ± 18.5	0.008

After establishing an optimal post-trigger serum hCG > 50mIU/mL, we evaluated which cycles were at risk to have sub-threshold levels under the sliding scale protocol. As expected, post-trigger serum hCG decreased linearly with increasing BMI. Using Spearman-rank correlation coefficients, there was a moderate-to-strong inverse linear relationship between BMI and serum hCG, and this correlation was statistically significant within each hCG dose group (*P* < 0.0001). The overall incidence of cycles with sub-threshold b-hCG was 2.3%. When stratifying by hCG dose and BMI, we considered cycles to be at risk if the incidence of sub-threshold b-hCG was greater than 10% for the respective group. As depicted in Table 2, b-hCG < 50 mIU/mL was more likely to occur in overweight patients receiving < 5,000 IU and obese patients receiving < 10,000 IU. Sub-threshold b-hCG in patients with a normal BMI receiving <10,000 IU was rare, and comprised only 0.8% of all cycles.

**Table 2:** Number (%) cycles with serum bhCG < 50 mIU/mL.

hCG dose (IU)	BMI (kg/m^2^)				
	<20	20–24.9	25–29.9	30–34.9	>35
10,000	0/794 (0%)	3/3091 (0.1%)	6/1230 (0.5%)	7/468 (1.5%)	14/337 (4.2%)
5,000	0/388 (0%)	8/1377 (0.6%)	29/507 (5.7%)	21/134 (15.7%)	30/77 (39.0%)
4,000	1/233 (0.4%)	27/822 (3.3%)	32/221 (14.5%)	9/51 (17.6%)	11/26 (42.3%)
3,300	2/176 (1.1%)	24/374 (6.4%)	16/89 (18.0%)	7/19 (36.8%)	2/3 (66.7%)

highlight: cycles determined to be at risk for b-hCG<50

IVF cycle characteristics according to hCG trigger dose were analyzed and are shown in “Table 3.” After stratification by SART age categories, there was no statistical difference in number of mature oocytes retrieved, fertilization rate, and number of embryos transferred with decreasing doses of hCG. Patients receiving lower doses of hCG had increased estradiol/MII ratio and blastocyst transfer across all age groups, which is consistent with an increased response to COS. Clinical pregnancy and live birth rates per retrieval were also analyzed according to hCG trigger dose. As shown in “Table 4” there was no difference in fertilization, clinical pregnancy or live birth rates across all hCG doses when administered according to the sliding scale protocol.

**Table 3:** IVF cycle characteristics according to hCG dose and patient age.

hCG (IU)	10,000	5,000	4,000	3,300
Age < 35 y				
# of patients	1,333	802	516	280
# of prior IVF attempts	1.9 ±2.0	1.8±2.0	1.5±1.6	1.4±1.7
# of mature oocytes	8.0±4.3	11.1±4.8	12.1±5.2	13.0±5.6
fertilization rate (%)	73.4±24.7	69.7±23.8	69.8±24.9	72.1±23.2
# of embryos transferred	2.1±0.6	2.2±0.6	2.2+0.6	2.2±0.5
E2/MII ratio *	186±139	226±210	260±225	312±327
Blastocyst transfer (%) *	16.3	24.8	31.9	33.6
Age 35–37 y				
# of patients	1,042	559	291	150
# of prior IVF attempts	2.3±2.1	2.2±2.1	2.1±2.0	2.1±1.8
# of mature oocytes	6.7±3.9	9.9±4.5	11.4±4.8	12.5±5.6
fertilization rate (%)	73.7±25.6	72.8±22.4	73.9±21.6	70.6±25.0
# of embryos transferred	2.6±0.9	2.7±0.9	2.8±0.9	2.7±0.9
E2/MII ratio *	221±164	240±147	271±201	327±295
Blastocyst transfer (%) *	9.0	18.9	23.7	26.8
Age 38–40 y				
# of patients	1,540	550	270	119
# of prior IVF attempts	2.9±2.6	2.6±2.1	2.5±2.3	2.4±2.2
# of mature oocytes	5.8±3.5	9.5±4.5	10.7±4.7	11.8±5.2
fertilization rate (%)	73.0±26.6	72.8±23.8	70.9±23.5	69.3±23.2
# of embryos transferred	3.0±1.2	3.3±1.2	3.4±1.1	3.2±1.1
E2/MII ratio *	235±158	260±174	293±190	369±395
Blastocyst transfer (%) *	5.8	14	15.3	22.2
Age 41–42 y				
# of patients	1,076	336	156	70
# of prior IVF attempts	3.1±3.0	3.0±2.4	3.1±2.8	2.5±2.2
# of mature oocytes	5.5±3.4	8.9±4.0	10.4±4.8	10.6±4.6
fertilization rate (%)	72.2±27.3	71.8±23.6	71.9±23.0	76.4±20.7
# of embryos transferred	3.1±1.5	3.8±1.5	4.1±1.5	4.1±1.4
E2/MII ratio *	254±183	290±229	337±364	357±220
Blastocyst transfer (%) *	4.6	9.7	9.5	10.4
Age >42 y				
# of patients	810	220	102	30
# of prior IVF attempts	3.5±3.2	3.2±2.3	3.1±2.4	2.8±3.1
# of mature oocytes	4.9±3.1	8.9±4.0	10.8±5.4	11.0±4.6
fertilization rate (%)	70.0±28.6	73.4±22.0	75.1±19.3	66.6±20.9
# of embryos transferred	3.1±1.7	4.5±1.8	4.8±1.8	4.7±1.8
E2/MII ratio *	256±171	294±251	326±333	319±183
Blastocyst transfer (%) *	2.8	7.6	10.6	6.7

* p < 0.05

**Table 4:** Clinical pregnancy and live birth rate per retrieval according to hCG dose and age.

hCG (IU)	10,000	5,000	4,000	3,300	p value
Age < 35 y					
# of patients	1,333	802	516	280	
Clinical Pregnancy (%)	43	49	48.4	44	NS
Live Birth (%)	39.2	45.3	46.7	43.8	NS
Age 35–37 y					
# of patients	1,042	559	291	150	
Clinical Pregnancy (%)	37	40.2	47.3	37.9	NS
Live Birth (%)	33	34.4	41.5	30.7	NS
Age 38–40 y					
# of patients	1,540	550	270	119	
Clinical Pregnancy (%)	31	37.6	32.3	28.6	NS
Live Birth (%)	25.7	31.3	27.8	23.5	NS
Age 41–42 y					
# of patients	1,076	336	156	70	
Clinical Pregnancy (%)	20	22	31.4	25.4	NS
Live Birth (%)	14.9	15.7	20.5	18.3	NS
Age >42 y					
# of patients	810	220	102	30	
Clinical Pregnancy (%)	8.8	10.9	16.7	6.7	NS
Live Birth (%)	5.7	6.8	10.8	3.3	NS

NS—not significant

Specific to high responders (E2 level at trigger > 3,000 pg/mL), a sub group analysis compared IVF outcomes between low dose hCG (3,300 IU) to a dual trigger with GnRH agonist and low dose hCG (2 mg leuprolide acetate + 1500 IU hCG). As shown in “Table 5,” peak E2 levels were significantly higher in the low-dose hCG group in patients younger than 35 years old. In addition, the number of mature oocytes retrieved was significantly higher in the dual trigger groups in all patients 42 and younger. In patients older than 42, there was no difference in oocyte yield between trigger types. Across all age groups, there were no significant differences in fertilization, clinical pregnancy, or live birth rates per transfer between trigger types.

**Table 5:** IVF outcomes per retrieval: Low dose hCG (3,300 IU) vs. dual trigger (2mg Lupron + 1500 IU hCG).

Age	<35		35–37		38–40		41–42		>42	
	3300	Dual	3300	Dual	3300	Dual	3300	Dual	3300	Dual
# of patients	355	131	197	76	182	72	88	35	61	26
Peak E2 (pg/mL)	*3,210± 555	2,977± 776	3,242± 572	3,229± 755	3,233± 479	3,268± 636	3,307± 586	3,174± 465	3,120± 563	3,332± 745
# mature oocytes	14±6	*18±9	13±5	*16±8	12±5	*15±7	12±5	*17±6	11±5	13±5
fertilization rate (%)	0.8±0.6	0.8±0.6	0.7±0.2	0.7±0.3	0.7±0.3	0.9±1	0.7±0.2	0.7±0.2	0.7±0.2	0.7±0.2
Clinical Pregnancy (%)	50	50	40	40	34	43	30	20	13	15
live birth (%)	43	40	32.5	30	26	33	17	11	7	4

* P < 0.05

Of the 10,427 fresh IVF-ICSI cycles meeting inclusion criteria, a total of 14 cases met our definition of moderate-severe OHSS. This represents an incidence of 0.13% over the 7-year study period. There were 2, 5, 4, and 3 cases of moderate-severe OHSS in the 10,000 IU, 5,000 IU, 4,000 IU and 3,300 IU hCG groups, respectively and their cycle characteristics are shown in “Table 6.” The rate of severe OHSS was 0.03% (n = 4 cases), of which two patients each were in the 4,000 and 5,000 IU hCG groups. All cases of severe OHSS showed evidence of laboratory abnormalities (transaminitis, electrolyte abnormalities, or hemoconcentration) and ascites requiring a paracentesis. All cases showed radiologic evidence of a pleural effusion, however only one was significant enough to require a thoracentesis. Inpatient treatment included close observation, intravenous fluids and anticoagulation, and all patients were discharged within 3 days. Of note, in the sub group analyses of high responders there was no significant difference in cases of moderate-severe OHSS (no cases in the dual trigger cohort vs. 3 cases in the 3,300 IU hCG group).

**Table 6:** Cases of OHSS in 10,427 IVF-ICSI cycles.

hCG (IU)	% (# of cases)	BMI (kg/m^2^)	Age	E2 at Trigger (pg/ml)	E2 post trigger (pg/ml)	# oocytes retrieved	% twins
10,000	14 (n = 2)	25.6 ± 2.4	34 ± 0	1823 ± 202.2	2434.5 ± 727.6	15.5 ± 6.4	100
5,000	36 (n = 5)	23.4 ± 2.3	35.4 ± 2.4	1931.6 ± 330.1	2780.2 ± 675.5	16.4 ± 5.2	60
4,000	29 (n = 4)	23.0 ± 0.8	31.8 ± 3.4	2572.3 ± 597.2	3169.5 ± 926.3	16 ± 1.6	25
3,300	21 (n = 3)	20.1 ± 1.8	34.3 ± 5.1	3009.3 ± 518.1	3607 ± 790	17.7 ± 3.8	0

## Discussion

The objective of the present study was to determine if lowering the dose of hCG trigger for high responders via a sliding scale protocol is an effective practice to induce oocyte maturity and evoke suitable luteal support, while decreasing the risk of OHSS. Our results show a 0.13% incidence of moderate to severe OHSS and .03% rate of severe OHSS in the context of conservative gonadotropin dosing, which are low overall when compared to published rates in the general literature. Post-trigger serum b-hCG > 50 mIU/mL appears to be a threshold for optimal oocyte maturity as b-hCG < 50mIU/mL was associated with significantly lower oocyte maturity and a subsequent 40.1% reduction in live birth rate even when controlling for age in multivariable logistic regression. Patients receiving lower doses of hCG had an increased response to stimulation evidenced by significant increases in estradiol/MII ratio and blastocyst transfer across all age groups. Consistent with a heightened response to stimulation, there was a non-significant trend of increasing number of mature oocytes retrieved. Despite response to stimulation, scaling hCG doses based on serum E2 level at the time of trigger showed equivalent fertilization, clinical pregnancy and live birth rates to the standard 10,000 IU hCG trigger.

There is mixed evidence that supports lowering the dose of hCG to reduce the incidence of OHSS. Although limited by small sample sizes, previous studies have compared 5,000 IU hCG vs. 10,000 IU hCG in high-risk polycystic ovarian syndrome (PCOS) patients as well as 3,300 IU vs. 5,000 IU hCG in high responders (E2 levels between 4000–5,500 pg/mL). These studies have failed to demonstrate a difference in the incidence of OHSS between their respective study groups [4] [19]. The potential impact of lowering hCG trigger doses was supported by Kashyap et al., who reported on their incidence of OHSS in 2,625 cases prior to and after instituting a similar hCG sliding scale to the one described here. Specifically, they reported a reduction of early and severe OHSS by factors of 7 and 4 respectively, solely as a result of lowering the hCG dose according to E2 levels at trigger [1].

Although lower doses of hCG may limit the incidence of OHSS, there is some concern that these doses may not provide a sufficient luteinizing effect for oocyte maturation. Evidence indicates that lower doses of hCG are as effective to the standard 10,000 IU with regards to achieving equivalent oocyte maturity, however no threshold of hCG has been established [15,19–21]. One study reported that patients receiving 2,000 IU hCG had a significantly lower number of MII oocytes retrieved compared to patients receiving 5,000 or 10,000 IU. The authors therefore concluded that the lower limit of hCG should be 5,000 IU [22]. The minimum dose of hCG was further challenged in a study of 94 high responders undergoing IVF-ICSI, which showed similar total and mature oocyte yields, fertilization rates, chemical and clinical pregnancy rates with 3,300 IU vs. 5,000 hCG [4]. Our study bolsters the aforementioned findings on a much larger scale, showing that hCG trigger with doses as low as 3,300 IU are sufficient to facilitate equivalent oocyte maturation, with the caveat that patients with higher BMI should potentially receive higher hCG vs. supplemental GnRH agonist trigger as they are at risk for post serum hCG levels below 50 pg/mL.

Recently there has been a paradigm shift to use GnRH antagonist-based protocols, especially in high responders, with subsequent GnRH agonist trigger as a means to eliminate early OHSS. In fact, although 57% (n = 5,919) of the cycles during the study period were agonist-based protocols, our institution has predominantly used antagonist protocols since 2009. The results of our sub-set analysis are consistent with literature showing that a dual trigger with a GnRHa and low dose hCG followed by aggressive estradiol and progesterone luteal support yields equivalent pregnancy outcomes to hCG trigger alone and may even result in increased oocyte maturity [9]. Given that some literature has shown reduced clinical pregnancy rates and increased first trimester pregnancy loss with GnRHa trigger, data regarding hCG trigger alone remains useful [14] [13].

To date, there is limited data regarding the pharmacodynamic properties of IM hCG as well as the serum threshold levels of b-hCG required for optimal oocyte maturation. Our study confirms the expected inverse relationship between b-HCG and increasing BMI. More importantly, this inverse relationship exists for each group of hCG trigger doses under our sliding scale protocol. A study investigating oocyte maturity in relation to b-hCG analyzed serum levels at 12, 36, and 84 hours post 10,000 IU IM hCG trigger in 404 patients [18]. The range of b-hCG levels 12 hours after trigger was 51–644 mIU/mL with a mean of 204 mIU/mL. They concluded that a serum level of 50 mIU/mL 12 hours post -trigger could be set as a threshold value for maximal oocyte maturity given that above this value, percentage of MII oocytes did not increase with increasing b-hCG concentrations. The study cohort contained no patients with b-hCG < 50 mIU/mL, thus making it difficult to set an accurate threshold value. Our data supports the above findings that a post-trigger serum hCG level of 50 mIU/mL may serve as threshold for optimal oocyte maturity. This threshold level may also be viewed as a saturation point as levels higher than 50 did not further increase % oocyte maturity.

This study is limited by its retrospective nature. Our study is the largest to date looking at the incidence of OHSS according to hCG trigger dose. In addition, by selecting only ICSI cycles we were able to assess absolute nuclear maturity with respect to serum b-hCG. OHSS remains a clinical spectrum with overlapping criteria for classification of severity and therefore potential under-diagnosis is a limitation of our study. However, since we do not perform routine outpatient draining procedures at our institution, we believe that the analysis of our hospital records accurately reflects our incidence of moderate to severe OHSS during the study period.

## Conclusions

OHSS remains the most significant complication of ovarian stimulation, however its incidence can be minimized by lowering the hCG trigger dose for high responders. Our study indicates that conservative stimulation with sliding scale hCG based on estradiol levels at trigger and fresh embryo transfer is associated with low rates of OHSS and favorable pregnancy rates. Doses as low as 3,300 IU hCG alone or dual trigger with 1,500 IU hCG and GnRH agonist are sufficient to maximize oocyte maturity in patients with a normal BMI. When hCG is administered alone, overweight and obese patients will likely benefit from higher doses > 3,300 IU due to risk of sub-optimal hCG absorption. Further research should investigate whether serum b-hCG levels < 50 mIU/mL after trigger is associated with sub-optimal IVF outcomes. The significant morbidity and potential perinatal complications of OHSS underlines the importance of primary prevention. Lowering the dose of hCG trigger is one important element of such prevention.

## Supporting information

S1 File 10247 IVF ICSI data file. Dataset of all 10,427 IVF/ICSI cycles included in the study.
(XLS)

S2 File Dual trigger data file. Dataset of sub group analysis comparing trigger with low dose hCG (3,300 IU) vs. dual trigger with GnRH agonist and low dose hCG (1,500 IU).
(XLSX)

S3 File OHSS data file. Dataset of the 14 cases of moderate to severe OHSS during the study period.
(XLSX)
